# Motor Imagery: How to Assess, Improve Its Performance, and Apply It for Psychosis Diagnostics

**DOI:** 10.3390/diagnostics12040949

**Published:** 2022-04-11

**Authors:** Alla Chepurova, Alexander Hramov, Semen Kurkin

**Affiliations:** 1 Center for Technologies in Robotics and Mechatronics Components, Innopolis University, 420500 Innopolis, Russia; a.chepurova@innopolis.university (A.C.); hramovae@gmail.com (A.H.); 2 Baltic Center for Artificial Intelligence and Neurotechnology, Immanuel Kant Baltic Federal University, 236016 Kaliningrad, Russia

**Keywords:** motor imagery, movement imagination, motor imagery assessment, transcranial magnetic stimulation, motor evoked potential, visual imagery, kinesthetic imagery

## Abstract

With this review, we summarize the state-of-the-art of scientific studies in the field of motor imagery (MI) and motor execution (ME). We composed the brain map and description that correlate different brain areas with the type of movements it is responsible for. That gives a more complete and systematic picture of human brain functionality in the case of ME and MI. We systematized the most popular methods for assessing the quality of MI performance and discussed their advantages and disadvantages. We also reviewed the main directions for the use of transcranial magnetic stimulation (TMS) in MI research and considered the principal effects of TMS on MI performance. In addition, we discuss the main applications of MI, emphasizing its use in the diagnostics of various neurodegenerative disorders and psychoses. Finally, we discuss the research gap and possible improvements for further research in the field.

## 1. Introduction

The ability to create and simulate new objects, sensations, and concepts in mind without directly affecting the senses is known as imagination. It is a complex phenomenon that is challenging to study, understand and explain. Publications in scientific journals dedicated to the experimental study of the essence of imagination appeared at the beginning of the 20th century [1]. Since then, thanks to the efforts of cognitive scientists, a rich theoretical framework has evolved that attempts to explain the nature of the imaginary. A crucial fact obtained by recent imagery research is distinguishing imagery types by representing what a subject tries to reproduce in his/her mind. Such differences exist between visual imagery (VI) and motor imagery (MI or kinesthetic imagery) [2]. However, both represent enthralling research areas, the current work dedicated mainly to the latter type of imagination.

Scholars have recently used “motor imagery” (MI) to refer to the imagination of moving particular body parts. Among the frameworks trying to explain the essence of motor imagery, there are two primary ones—motor simulation and motor emulation theory [3]. The Motor Simulation Theory (MST) [4,5] provides a constructive explanation of the relation of imagery tasks such as motor imagery (MI task), observation, and the intention of motor tasks to motor execution (ME) tasks itself. According to MST, motor images have the same properties as analogous motor representations and thus have the same functional relationship to the perceived or represented movement and the same causal role in its development [4]. Alternatively, according to emulation theory, to simulate mechanical movement, proprioception, and kinesthesis, the “forward model” is represented by motor commands that drive body/environment emulators, which are motor and sensory representations. In such a way, coupled motor and sensory systems form a complex process of emulation that provides an adaptive motor control mechanism for ME. While performing MI, the motor system is coupled with afferent sensory systems, which provide the sensor feedback without input for MI [6].

To summarize, MST supposes a rehearsal of the motor system influenced solely by internal motor representations, while emulation theory makes claims about the emulation of both the motor and sensory systems simultaneously. Numerous studies have been carried out to investigate which theory provides a more plausible explanation for the phenomenon of imaginary movement or provides a novel cause of imagery inner mechanisms [3,7,8,9]. This resulted in many papers on empirical comparisons of ME, MI and VI using different tools (fMRI, EEG, MEG, TMS, EMG) and approaches (behavioral chronometry, connectivity analysis, different statistical techniques). However, what is appealing in both these theories is that they provide a rich framework for supporting the effects of mental training, observational learning, and the use of neuroprosthetic devices for people with motor disabilities. As for the last point, these theories served as a prerequisite for creating a vast number of different Brain-Computer Interfaces (BCIs) that can discriminate the different types of imagery motions or intentions of users online and provide alternative ways to interact with the environment [10,11].

In this review, we provide a comprehensive observation of the recent results in the field of imagery movements by grouping all the empirical results achieved by different tools and approaches together, providing an inner-comparison (between results achieved in the same way, e.g., by the same instrument) and an outer-comparison (comparing results from various sources) and critically assessing them to gain a more complete and systematic picture of human brain functionality in cases of MI. Recent relevant papers were aggregated and formed brain region maps for MI and ME and a comparison table of grouped studies dedicated to neuroimaging during MI and ME. Recent studies that use single-pulse or repetitive transcranial magnetic stimulation (spTMS/rTMS) were analyzed to expand the results of the neuroimaging section. These methods are active and more dynamic in terms of neurofeedback than those presented in the first stage of the search (fMRI, EEG, et al.). Considered studies with TMS added complementary details to the difference between ME and MI obtained with TMS motor-evoked potential (MEP) analysis and revealed the neuroplastic potential of the MI + TMS protocol.

Since MI has great applications (for developing BCIs [12,13], rehabilitation tasks [14,15], prediction and prevention of neurodegenerative disorders [16,17,18,19], psychosis investigation and diagnostics [20], sports [21,22]), we also discuss an important issue related to investigating the possibility of improving MI performance using external influences on the brain in the form of TMS. Moreover, we deemed it necessary to review the MI assessment methods found in the selected studies, which resulted in an overview table with descriptions of frequently encountered assessment methods that may help researchers in setting a protocol for future studies. This results in a better understanding of the nature of imagery movements that can help for the future elaboration of motor imagery theories and provide a proven description of physiological processes underlying motor imagination as a reference model for imagery assessment in future research. In addition, we discuss the main applications of MI, emphasizing its use in the diagnostics of various neurodegenerative disorders and psychoses.

## 2. Materials and Methods

### 2.1. Conducting Literature Review

For this review, published papers were retrieved in the scientific databases using the Google Scholar search engine. The review process was divided into two parts dedicated to different aspects of MI studies.

For the first part of the review dedicated to MI/ME brain areas mapping, we used the following terms in search query construction: “motor imagery”, “mental imagery”, “imagery movements”. After a primary search on these terms, we cross-checked for omitted words and formulations plausible for further search queries in the texts of previously found papers.

Afterwards, we refined previously defined MI terms for a more sophisticated search dedicated to TMS investigation and searched in the database with a query “motor imagery” “TMS”, where double quotes stand for necessarily included terms in the resulting papers. At both stages of the search, works were considered relevant for the review if we found one of the search queries or equivalent reformulation in its title, abstract, or keywords.

#### Inclusion Criteria

To give a comprehensive picture of the topic, we applied several criteria to sort out papers with the undesirable publication type, citations number, and other standard results of the search. We used only primary sources and neither secondary sources nor grey literature. However, there was a selection criterion among primary sources, too; we included only published papers and reviews.

The following addition to the protocol mentioned above was used in the first stage of the review to cover a more general picture of the MI area. The journal of publication should have an impact factor greater than 2.5 at the moment of publication, or the number of citations should be greater than 100. There was no strict constraint on the year of publication, as EEG- or fMRI-based experiments have been available and widely conducted since the 1990s. More importantly, the earliest consistent results, which are still mentioned in recent studies, were achieved in these years.

As for the second stage of the review conduction covering TMS studies, we had a different purpose. The following additions to the primary protocol were made to cover recent tendencies of TMS use in MI research. The year of publication should have been 2017 or later; therefore, due to the constraints of publication year, a paper should have at least 10 citations at the moment of review conduction. Some papers which are similar to the works mentioned in the resulting table of the TMS stage and did not fit one of the criteria were mentioned in the text of the Results and/or Discussion sections supporting the findings of recent results.

### 2.2. Data Collection and Analysis

After the stage of the selection process, we conducted a data collection process. For the first part of the review dedicated to the generalization of the MI/ME brain region maps, we tried to avoid including studies with subjects with any neurophysiological pathology. Although several selected papers contained results for heterogeneous groups due to the experiment design, for this review, we extracted information about control groups only, i.e., subjects that are healthy and not trained for MI. As for the part of the review dedicated to recent TMS studies and applications, we did not apply any additional restrictions to the experiment protocol trying to cover all the trends that appeared in the MI-TMS area several years ago.

Further work on the collected studies led to the decision on the type of analysis. Most of the endorsed papers had a quantitative method of investigation with homogeneously reported findings. Therefore, we have chosen the type of review with elements of meta-analysis. Meta-analysis is a systematic study of the literature on a specific issue that yields a numerical assessment of the impact of a treatment technique or exposure. The comprehensive summarizing of scientific domains used in meta-analysis has emerged as a more formal, repeatable, and rigorous approach to evidence aggregation.

After review conduction, we also aggregated MI assessment methods that were used in selected studies. It resulted in a table with assessment methods classification, its main idea, and possible disadvantages.

## 3. Results

### 3.1. Generalization of Brain Regions Involved in MI and ME

**Figure 1:**
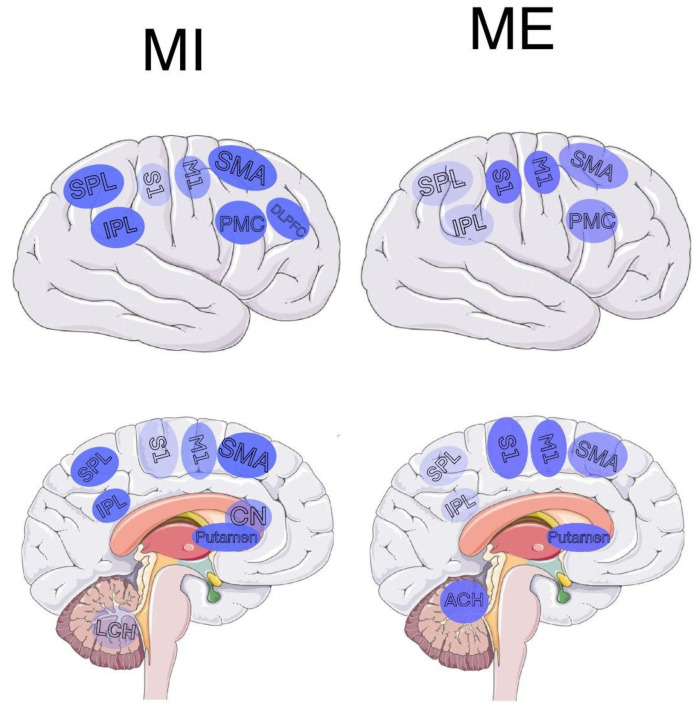
Activation maps of brain regions during MI and ME. (Left) Brain activation map for MI; (right) brain activation map of ME. Abbreviations: PMC—premotor cortex, M1—primary motor cortex, S1, S2—primary and secondary somatosensory cortices, SMA—supplementary motor area, CN—caudate nucleus, LCH—lateral cerebellar hemisphere, ACH—anterior cerebellar hemisphere, SPL—superior parietal lobe, IPL—inferior parietal lobe, DLPFC—left dorsolateral prefrontal cortex. The intensity of color depicts the strength of activity in the corresponding region.

Studying and systematizing collected data to compare MI and ME emerged results represented in Table 1 and Figure 1. There is a significant overlap of the brain regions responsible for MI and ME. This similarity in brain activation for MI and ME could be explained by belonging to the same motor representation system [23]. Nevertheless, even though most research in this field is focused on activation sites overlap, a more comprehensive observation indicated significant differences between motor imagery and physical execution (see Section 4).

### 3.2. Motor Imagery Assessment

Imagination is a complex phenomenon in which vividness, intensity, and representation are not the same in every person [40,41]. Studies show that some people cannot even use various types of imagination, including motor imagination [41]. The difference from person to person in terms of forms of imagination makes the research process in this field complex. Therefore, assessing imagery ability prior to conducting an imagery experiment or participating in an imagery training program is essential.

For creating a successful method for assessing MI, it is crucial to understand its properties and aspects that could be expressed numerically and evaluated for a subject. According to Jeannerod [42], motor representations are involved in both conscious and unconscious cognitive activities. Examples involving conscious motor representation are imagining a limb movement in the first person and dreams, so-called explicit motor imagery. In contrast, examples that involve unconscious motor representations are prospective action judgments and motorically driven perceptual decisions (e.g., defining hand laterality in different depicted positions), so-called implicit motor imagery. Jeannerod also distinguished motor imagery from dynamic visual imagery and movement imagery from an external viewpoint, referring them to a visual type of imagery. The essential part of motor imagery that distinguishes this type of imagery from a visual one is a kinaesthetic sensation that allows the subject to experience the feeling of performing movements. Nevertheless, the phenomenon of motor imagery could not be reduced to the kinesthetic sense only as it develops in a body-centered and visuospatial context. So, motor imagery includes kinesthetic, visual, and spatial aspects of movement executed by an imager [43]. Therefore, all of the above aspects as characteristics of the experiencing of imaginative movement must be considered when assessing motor imagination.

The essential MI assessment techniques exploited in experimental studies were aggregated in Table 2. Moreover, original papers that introduced these assessment methods were cross-checked for additional references for other types of techniques compared to them. The methods were grouped by type of MI, explicit or implicit, by type of task subjects required to perform in these tests.

### 3.3. Transcranial Magnetic Stimulation in MI Research

Transcranial magnetic stimulation (TMS) is widely accepted as a powerful noninvasive tool for analyzing the central and peripheral nervous systems of people. Magnetic stimulation has a similar activation mechanism to electrical stimulation for activating peripheral nerves. TMS excites the pyramidal neurons transsynaptically resulting in the rise of I (indirect) waves. In contrast, transcranial electrical stimulation excites the pyramidal tract axons directly, either at the beginning segment of the neuron or at proximal internodes in the subcortical white matter, resulting in D (direct) waves [61].

**Figure 2:**
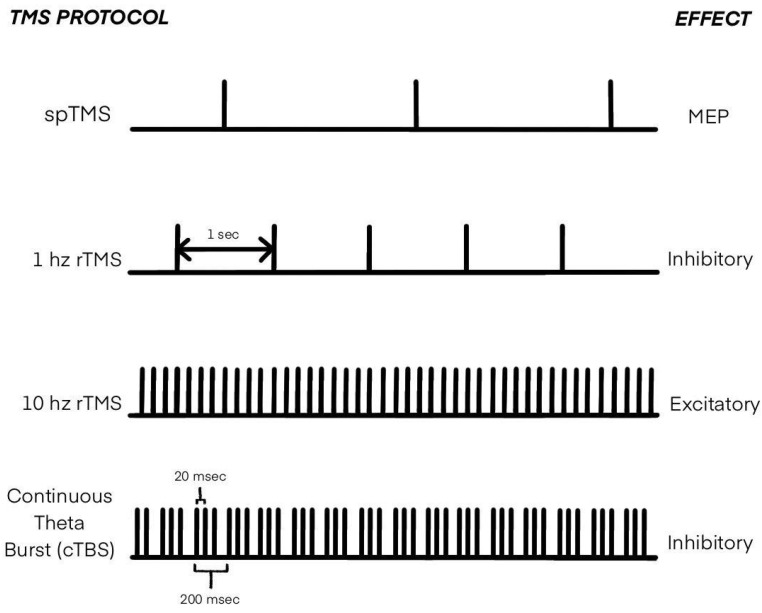
Types of transcranial magnetic stimulation (TMS) applied in MI research.

The significant and well-studied feature of TMS is its application in motor and motor imagery studies. Figure 2 demonstrates the most commonly used TMS protocols in MI research. TMS applied to the motor cortex induces an excitatory effect on corticospinal neurons, which can be assessed with electromyography as motor evoked potentials (MEPs) and/or [61]. Moreover, cortical stimulation with TMS can induce not only excitatory effects but inhibitory too. This characteristic may be utilized to analyze functions of the brain regions other than the motor cortex. It results in the capability of TMS to map brain regions and investigate functional connectivity among distinct cortical regions [61]. Another crucial feature of TMS is its long-term effect on brain function. Even after stopping the stimulation, the corresponding stimulating effect lasts for a long time (starting from several hours to several months in the case of medical applications [62,63,64]). All these features make TMS a powerful tool for motor imagery studies.

While the studies revealed in Section 3.1 correspond to most cited works dedicated to activated brain areas during MI and ME, they all exploited fMRI/MRI/EEG/MEG as a tool for brain activation analysis. However, the study of motor cortex properties during MI or ME is not limited to using these measures alone. Although fMRI, EEG, and MEG can depict the brain more fully in terms of its areas and activity in them than TMS, TMS stands out as a more “dynamic” active tool for MI study due to its properties. Single-pulse TMS (spTMS) provides measurable neurofeedback, resulting in the possibility of using it in a more precise so-called neurophysiological MI assessment. At the same time, repetitive TMS (rTMS) allows the study of the importance and functional connectivity of different brain areas in MI experiments [61]. Moreover, inhibitory and excitatory rTMS coupled with imagery activity could result in a neuroplasticity effect. TMS reinforces MI potential in applications such as stroke rehabilitation, brain-computer interfaces (BCIs), and motor learning. Given that, the separate section was dedicated to reviewing the subject of TMS utilization in MI research and its applications.

#### 3.3.1. spTMS in MI Research

Single-pulse transcranial magnetic stimulation (spTMS) is non-invasive technique for examining and modulating the excitability and plasticity of the human brain. Single-pulse TMS (spTMS) elicits the complex activation of various types of cortical neurons in the motor cortex, resulting in a stereotyped instantaneous response in muscle termed excitatory muscular motor evoked potential (MEP) [61]. Most studies examining high-frequency rTMS’ post-train effects have assessed MEP amplitude in response to a single spTMS train [65,66,67,68,69,70,71]. The majority of these trials demonstrated an instant increase in excitability. In turn, this section reports the results of the last studies investigating MI’s post-train effects on spTMS evoked MEP amplitude. Grouped data extracted from papers are presented in Table 3. Nine papers met the criteria described in the Methods section.

#### 3.3.2. rTMS in MI Research

Repetitive TMS (rTMS) is a non-invasive brain stimulation technique whose effect on the subject varies by stimulation settings. High-frequency (>1 Hz) stimulation is thought to cause a local increase in cortical excitability, whereas low-frequency (≤1 Hz) stimulation causes the opposite effect [81,82]. A significant number of physiological studies investigating the effects of rTMS have been performed in recent years to support these claims. These works are divided into two categories: those focusing on cortical excitability and those studying cortical inhibition. In this subsection, we review both the inhibitory and excitatory effects of rTMS applied to MI research and its utilization to reveal recent tendencies. The results of the selected paper analysis were aggregated in Table 4. Overall, there were eight papers chosen that corresponded to the criteria described in the Methods section.

## 4. Discussion

### 4.1. General Conclusion

First of all, we clustered all the selected papers by the brain areas activated during the different types of movements to find the most plausible and frequently observed results on this matter. As a result, we have obtained three characteristic brain maps—for MI, ME, and common for MI and ME. These results could help us to better understand the nature of imagery movements that is useful for future elaborations of motor imagery theories and provide proven descriptions of physiological processes underlying motor imagination as a reference model for future assessment of imagination in the new research. In turn, it could profit from a broad field of research, including sports, music, disease prevention, and rehabilitation [14,22,91,92,93,94].

Next, we systematized the known MI assessment methods and discussed their limitations. Therefore, one can consider the results of this analysis to find the recently used imagery assessment methods appropriate for the planned experiment. Consistent use of the same assessment methods reduces research variability and improves reproducibility, so it is important to generalize assessment methods for more reliable results.

Finally, we have analyzed the recent tendencies in MI studies with TMS. As a result, we grouped the studies by their goal, methodology, and results. Revealing such findings, we defined the research gap and summarized current findings and limitations in this field.

### 4.2. Generalization of Brain Regions Involved in MI and ME

#### 4.2.1. Common Sites for MI and ME

Notably, there are overlapping activation sites associated with both motor imagery and physical execution in the premotor cortex (PMC), the primary motor cortex (M1), the primary (S1) and secondary (S2) somatosensory cortices, the supplementary motor region (SMA), striatum (which is made up of the caudate nucleus and the lentiform nucleus, including putamen), cerebellar areas, the inferior, superior, and frontal-parietal lobes.

#### 4.2.2. Specific for MI Sites

Although previously described regions are common for both MI and ME, SMA [34], frontoparietal lobe [29], and left posterior parietal lobe [29] demonstrate stronger activation during motor imagery. Moreover, common for MI and ME striatum activates more strongly during MI in the area of the caudate nucleus [11]. Lower activation was observed in M1 and particularly low in S1, S2, and the anterior cerebellar areas [34]. The lack of somatosensory input could explain these differences during MI. The involvement of the posterior cerebellum in MI depends on the degree of acquisition of motor imagery and becomes higher with real motor execution practice. A possible explanation is the lack of sensory input for MI while not having enough practice and more precise and embodied representation otherwise [95]. Furthermore, the MI-specific areas mentioned in the reviewed papers are rostral premotor, central sulcus, and frontal gyri [22].

Notably, only MI recruited the left dorsolateral prefrontal cortex (DLPFC) [39]. The DLPFC is implicated in frontal-executive functions related to action preparation [96], which is believed to have similar neural substrates to MI. Moreover, DLPFC plays a role in movement inhibition [97], preventing overt movement during MI.

#### 4.2.3. Specific for ME Sites

Common for motor imagery and execution, M1, S1, S2, and cerebellar areas exhibit stronger activation and a more prominent contralaterality for ME. Low activation for ME was observed in SMA, posterior, and inferior parietal lobes. The ME-specific area mentioned in the reviewed papers is precentral gyri.

### 4.3. MI Assessment

Methods for motor imagery assessment used in MI studies covered by the current review were grouped in Table 2. Such a variety of methods creates potential inconsistency in MI studies protocols and, more importantly, could alter the results. So, a plausible solution would be an introduction of a universal and commonly accepted protocol of imagery assessment. Speaking of the general method of evaluation, it is necessary to choose one that covers all aspects of MI, both visual and kinesthetic, suitable for a broad audience of both healthy people and those with disabilities, not requiring the performance of domain-specific movements or any prior knowledge of the movements performed.

Considering all the abovementioned criteria, the Questionnaire upon Mental Imagery (QMI) is not the option due to its general nature, assessing overall imagery ability rather than the motor one. On the contrary, the Florida Praxis Imagery Questionnaire (FPIQ), the Movement Imagery Questionnaire (MIQ), the Sport Imagery Questionnaire (SIQ) and the Exercise Imagery Questionnaire (EIQ) do not fit the criteria of a universal method because of the specificity of the task to be performed by subjects. The Vividness of Visual Imagery Questionnaire (VVIQ) and the Vividness of Movement Imagery Questionnaire (VMIQ) are not appropriate choices due to the lack of measurement of the kinesthetic aspect of MI. Remained MIQ-2 and the Kinesthetic and Visual Imagery Questionnaire (KVIQ) are the most appropriate options among questionnaire-based assessment methods for healthy subjects. MIQ-RS having a significant correlation with KVIQ [54] is a better option for heterogeneous populations. Among other non-questionnaire methods, only the Hands Laterality Judgement Task (HLJT) and mental chronometry tests, including simple tasks, meet the criteria. However, both methods should be used considering the variation in the timing of tasks independent of the subject’s MI ability. A suitable solution could be coupling these methods with dRT (the difference in reaction time) to consider differences between time values before and after practice rather than the absolute value of time.

The scientific community is already taking steps toward more uniform and easy-to-use assessment methods. Thus, the software developed in [98] uses the dRT paradigm to provide an easy-to-understand interface for performing Implicit Sequence Learning (ISL) paradigm assignments by subjects and evaluating results by researchers. Findings in [98] demonstrated a significant correlation between KVIQ score and dRTs, revealing the connection between imagery ability and the ability to learn MI. Still, limitations persist, and improvements in this domain could significantly speed up and improve the results.

### 4.4. Transcranial Magnetic Stimulation in MI Research

#### 4.4.1. spTMS

There are several tendencies regarding MI research with spTMS (see Table 3). Correlation between MEP amplitude and MI score assessed by standard questionnaires is one of the recent directions in spTMS-MI studies. One study included in the aggregated table [72], and a well-cited one that does not fit the requirement of a year of publication but is worth mentioning [99], used similar protocols for spTMS and assessed MI quality in different ways. The first one revealed an association of greater MEP amplitude during MI with more vivid kinesthetic images assessed by the vividness of movement imagery questionnaire (VMIQ-2) and faster motor reaction. The second one demonstrated a positive correlation between MI-adopted visual analogue scheme (VAS) score and corticospinal excitability during MI coupled with action observation (AOMI paradigm). The conclusion of the relationship between MI vividness and MEP amplitude is consistent with the findings of both studies.

However, this is not the only study among those selected that included the AOMI paradigm. A whole series of recent studies [73,74,75] used the spTMS-MEP protocol to investigate aspects of MI in various settings. One of these, ref. [75], is outstanding in terms of the aim of the study—trying to counteract a decrease in corticomotor excitability (CE) specific to musculoskeletal pain with AOMI practice. These findings revealed that reduction in CE was compensated by practicing the AOMI task while in pain, as demonstrated by the fact that TMS-MEPs did not alter throughout the AOMI + PAIN session. However, the results of the other two AOMI studies [73,74] are not as clear-cut. Findings in [74] suggest AOMI as such, but not independent MI or AO, enhanced corticospinal excitability, thus contradicting the conclusions of [73], implying AO does not appear at the level of PMC, and MI is sufficient for the explanation of the AOMI facilitatory effect. However, the well-cited paper [100] reported that the kinesthetic aspect of MI, but not the visual aspect, is responsible for corticospinal excitability, supporting the conclusion of [73].

Another common MI aspect studied by recent studies using spTMS is neuroplasticity. References [76,77] demonstrated that MI training sessions could induce plastic changes in the motor system as a correlation of MEP amplitude with a measure of motor cortical adaptation shown. Reference [78] also compared MI-induced plastic changes with ME-induced ones and came to the conclusion of their similarities, yet revealed the fact that MI-induced changes demand more trials to appear than ME ones. Moreover, experiments in [79] showed that the MI-induced plasticity, revealed in the studies above, is use-dependent as TMS-induced movements proportionally deviated to the trained direction opposite the dominant one.

It is worth mentioning another study concerning the application of spTMS determining the MI role in cortical processes. In experiment [80], two groups of subjects received different instructions during MI: the experimental group was instructed to avoid overt movement during MI, while the control group was not. Results demonstrated an inhibitory effect of pure MI proven by the decreased TMS-evoked MEP amplitude in the control group.

#### 4.4.2. rTMS

The emerging results (see Table 4) demonstrate several prevailing trends in the rTMS study paradigms. The first one is using rTMS inhibitory stimulation to study the involvement of different cortical motor areas in MI or its specific subtypes such as kMI (kinesthetic Motor Imagery) or vMI (visual Motor Imagery) of healthy subjects. These studies [83,84,85] indicate which areas are crucial for MI skill acquisition; therefore, weakening or damaging these areas results in reduced MI ability, vividness sensation during MI practice, and difficult MI learning. Notably, the results of these studies also demonstrate a deeper difference between MI and ME. Thus, the findings of these works are crucial to consider in MI training both for BCI, for a more precise distinction of activities by classifying algorithms, and for various kinds of motor rehabilitation to determine the individual MI ability of the patient and therefore the prospects for recovery when using MI techniques.

Thus, results in [83] allow an understanding of the roles of S1, MI, and dPMC in the MI process. In particular, dPMC and S1 stimulations reduce and facilitate corticospinal excitation (CSE) during kMI, respectively, while M1 stimulation does not alter muscle-specific facilitation in kMI settings at all. In [84,86], different experiment designs were used: theta-burst TMS stimulation and rTMS stimulation, respectively. However, in both cases, stimulation was applied to the inferior parietal lobe (IPL), which allowed us to find out whether IPL is a crucial part of MI mechanisms and refine the role of the IPL in the MI process. Thus, these studies demonstrated the IPL’s role in visuospatial functions of MI activity, such as controllability and visual manipulations.

Speaking of BCI in rTMS MI studies, it is noticeable that it is also one of the predominant areas among the included studies devoted to the analysis of post-stroke patients. Among BCI and rTMS-based studies, there are also protocols involving virtual reality (VR), where subjects need to perform the same MI activity but with feedback in the form of VR simulation. Studies [89,101] exploited similar protocols, including BCI-VR training combined with inhibitory rTMS stimulation over non-stroke M1. They reported consistent findings regarding a significant level of motor improvements in stroke patients for the rTMS + BCI-VR protocol compared to the control BCI-VR one. Conversely, BCI studies, including excitatory rTMS over the impaired hemisphere, focused more on BCI classification score improvement. Both [90,102] reported ME and/or MI classification scores improvements.

### 4.5. MI Learning

As the TMS studies revealed [77,78,79], MI indeed can cause plastic changes similar to those observed during physical training. Moreover, these plastic changes can be controlled by the direction of the learned task, as was shown in [79]. These observations prove the validity of the plausible motor mental training framework. The training MI protocols used in spTMS studies can be coupled with rTMS inhibitory application over the hemisphere opposite to the one that needs excitatory stimulation or rTMS excitatory stimulation right over the hemisphere that needs excitation to boost MI learning [87,88], which is especially important for stroke subjects.

Coupled MI training and additional rTMS stimulation also demonstrated significant improvements in the classification metrics of BCI algorithms [89,90], which implies the better acquisition of MI tasks associated with the better distinguishing of MI representations with the BCI embedded algorithm. This finding could be used as an additional instrument for MI learning assessment. Numerical representation of improvement associated with the direction and intensity of learning can help subjects to effectively consider interface feedback by directing their focus and efforts in the direction of learning that can bring greater efficiency gains in the accuracy of the algorithm. This assumption is supported by experimental studies [103] that demonstrated the potential of a visual neurofeedback framework in MI training for the swallowing task. Unfortunately, in [103], fMRI visualization was used for neurofeedback, making this approach less scalable and affordable. So, further investigations on other neurofeedback visualization techniques are needed to provide a generalized procedure controllable by subjects.

Another direction of research on MI-based learning is using VR technologies in the experiment setup as was done in [89]. It can save time for experimental setup and provide subjects with more familiar and natural feedback, similar to what they are used to encountering in real life when performing the same ME tasks, therefore facilitating the MI learning process.

### 4.6. Applications, MI for Diagnostics

The applications of MI for BCI operation, in rehabilitation systems in sports, have become conventional. There is a bulk of papers and reviews on these issues [12,13,14,15,21,22]. Another exciting and promising trend in MI applications is researching and diagnosing various neurodegenerative diseases and psychoses, especially schizophrenia [16,17,18,19,104]. For example, based on the analysis of the difficulties in MI, the authors of the review [20] conclude that schizophrenia involves, as well, impairments of the posterior parietal cortex. Moreover, they present a novel hypothesis that suggests differential impairments of the left and right parietal cortices in schizophrenia, which may help explain many of the first-rank symptoms of the disorder. The study [105] revealed that patients with schizophrenia performed motor imagery of gait slower than healthy controls. This deficit could be in part explained by impaired executive function and specifically by a disturbance in the sensitivity to interference. The paper [106] showed that schizophrenia patients, similarly to nonclinical participants, overestimated tool-related benefits and underestimated tool-related effort in terms of time when they mentally simulated a task requiring the use of a tool. These results open new perspectives on the issue of effort in schizophrenia.

The work [107] investigated motor retardation in bipolar depression. FMRI showed that, during motor imagery, the patients activated the posterior medial parietal cortex, the posterior cingulate cortex, the premotor cortex, the prefrontal cortex, and the frontal poles more than the healthy controls did. In addition, limbic and prefrontal regions associated with self-reference and the default mode network were altered during motor imagery in bipolar depression with motor retardation. The study [108] investigated the influence of unipolar depression on MI ability using a pointing task. Compared to controls, depressed patients showed marked motor slowing on actual and imagined movements. More significant temporal discrepancies between actual and mental movements were observed in depressed patients than in healthy controls. Furthermore, depressed patients modulated, to some extent, mental movement durations according to the difficulty of the task, but this modulation was not as strong as that of healthy subjects. These results suggest that unipolar depression significantly affects the higher stages of action planning and points out a selective decline of motor prediction.

Reference [109] addressed action simulation processes in adolescents with Asperger syndrome (AS) using the following MI tasks: the classical hand laterality task and the mental rotation of letters. The authors demonstrated a specific alteration of motor imagery skills in AS—they found the biomechanical effect (the advantage for judging hand pictures showing physically comfortable versus physically awkward positions) in typically-developing participants but not in participants with AS.

Thus, motor imagery is a powerful and promising tool for research and diagnostics of various neurodegenerative disorders and psychoses, whose potential has not yet been fully realized.

### 4.7. Current Research Gap

One of the identified gaps in the field of motor imagery is a lack of emphasis on the individual characteristics of the subjects and experiment conditions. Researchers tend to conduct studies in heterogeneous groups in which there is little or no regard for factors such as age, dominant hand, current health, mental conditions, and motivation. So that a unified or average view of the MI representation is created, which brings a little for the use of MI in practical applications. Recent studies demonstrated age-related changes in ME representations implying possible changes in MI due to the overlapping brain representations [110]. Thus, more attention should be paid to the context of the experiment from the subject’s view. Additional criteria, such as experience with MI and cognitive capacity, should be considered for both recreational and clinical applications. Prior to the experiment, researchers also need to consider supplementary aspects, such as the subjects’ physical condition, age, and motivation. In this way, it would be better to gain information about how these aspects affect the success of motor imagery practices.

Another essential aspect in the MI field that further research may need to study is an objective assessment of the imagery ability of subjects. Revealing more individualized biomarkers that indicate MI also implies gaining knowledge of features that characterize expertise in MI, which would result in a more objective assessment of the subject’s overall ability to imagine and the quality of the process of acquiring imagery skills. Finding this will make empirical research in MI more subjective, significantly advance our knowledge of the imagination and provide a basis for new research in MI. A potentially successful direction in addressing this issue could be the quantized measurement of MI capability, such as with spTMS pulses. As the studies have shown [83,84], there is a significant relationship between MEP amplitude and scores in MI questionnaires and the dRT paradigm, suggesting the possibility of using the method as a more accurate determinant of MI ability.

Further, one can utilize the knowledge of expertise-specific features to accelerate acquiring imagery ability. This could be done via rTMS stimulation applied to the specific brain sites that are responsible for expertise in MI, which would provide a chance for poor imagers, who are often not considered and are even weeded out in MI studies, to gain skills in MI and take full advantage of its recreational opportunities.

Another important aspect that is often overlooked in MI research is the quality of MI performance without overt action by subjects. In the protocol of each MI study, it is crucial to introduce EMG control of unconscious movements or provide direct instructions to subjects to avoid overt movements. Differences in such details between study protocols create variability in results and lead to unintelligible findings [73,74,80].

## 5. Summary

This review aimed to generalize studies of different aspects of MI, including the influence of transcranial magnetic stimulation. The essential issue that still remains in this field is the absence of a universal criterion of imaginary movement, not averaged within the group but applicable to each object within it. We might accurately assess effects through heterogeneous areas of endeavor to advance the existing motor imagery practice, accelerate the development process of imagery skills and expand the audience to which the recreational opportunities of MI training will be applicable if we were able to define generalized biomarkers of motor-imagery based learning processes and MI expertise-specific features of brain activation.

## Figures and Tables

**Table 1:** Brain areas activated during MI and ME, aggregated papers.

Brain Area	Common for MI and ME	MI	ME
Inferior parietal lobe	[24,25]	[24,25,26]	[24,25]
Superior parietal lobe	[25,27,28]	[25,26,27,28]	[25,27,28]
Posterior parietal lobe	[29]	[29,30,31]	[29]
Frontal parietal lobe	[11,23]	[11,23,29]	[11,23]
Prefrontal cortex	[10]	[10]	[10,11]
Subcortical	[11]	[11,25]	[11]
Rostral premotor	-	[11,31]	-
Striatum	[11,24]	[11,24,26,32,33]	[11,24]
Cerebellar areas	[10,11,23,25,29,34]	[11,23,25,29]	[10,11,23,24,25,26,27,28,29,33,34]
M1	[25,28,34,35]	[25,28,34,35,36,37]	[10,23,24,25,28,29,33,34,35,37]
S1	[28,34]	[28,34]	[10,28,29,34,37]
S2	[38]	[38]	[38]
SMA	[10,27,28,34]	[10,25,27,28,29,34]	[10,23,24,27,28,34]
PMC	[28,34]	[28,34]	[26,28,34]
Central sulcus	-	[11]	-
Precentral gyri	-	-	[35]
Frontal gyri	-	[29]	-
Left DLPFC	-	[39]	-

**Table 2:** Motor imagery assessment techniques.

Type	Name	Paper	Main Idea	Limitations
Explicit motor imagery				
Self-report questionnaires	QMI (Questionnaire Upon mental Imagery)	[44]	General vividness if imagery	Too general to be reliable measure of MI.
	VVIQ (Vividness of Visual Imagery Questionnaire)	[45]	Measuring the vividness of visual imagery	It is not enough to measure only the visual aspect of MI; it is a complex type of imagery that includes kinaesthetic sensations too.
	VMIQ (The Vividness of Movement Imagery Questionnaire)	[46]	Analysing visual and kinaesthetic aspects of MI, based on previously used VVIQ [45]	Measuring more visual aspects of MI, rather than kinaesthetic; no mentions of kinaesthetic aspects in instructions; rating scale is anchored to the visual sensations.
	MIQ (The Movement Imagery Questionnaire)	[47]	Measuring visual and kinaesthetic aspects of MI; two scales—visual and kinaesthetic	Specific and complex movements.
	MIQ-2	[48]	Measuring visual and kinaesthetic aspects of MI; two scales—visual and kinaesthetic, more easier for performing movements	Perfectly healthy subjects are needed.
	FPIQ (Florida Praxis Imagery Questionnaire)	[49]	Measure of imagery of practiced movements	Too specific, rarely used, no physiological evaluation.
	SIQ (Sport Imagery Questionnaire), EIQ (Exercise imagery Questionnaire)	[50,51]	Measure of imagery of specific sport-related movements	Even more specific than FPIQ; only for athletes testing.
	VAS (Visual Analogue Scheme)	[52]	Visual Analogue Scheme was adopted for MI assessment to describe the level of MI vividness	Initially VAS was not constructed for MI assessment, need refinements for different MI task adoption.
	KVIQ (The Kinesthetic and Visual Imagery Questionnaire)	[53]	The ordinal scale of five points representing the individuals’ ability to imagine the clarity of the image (visual: V subscale) and the intensity of the sensations (kinesthetic: K subscale) from a first-person perspective	Limited to assessing persons with physical disabilities, it does not take into consideration lesions that may impair the ability to image.
	MIQ-RS	[54]	Incorporates both the visual and kinesthetic aspects of mental imagery and the correlation between motor imagery scores and degree of impairment	Unlike KVIQ, MIQ-RS is appropriate for both healthy and disabled groups of people; still, it does not take into consideration lesions that may impair the ability to image.
Explicit motor imagery				
Mental chronometry paradigm	Mental chronometry tests	[55]	Analyzing the time that motor imagery takes for a subject; based on the assumption that real execution time reflects imagery execution time, therefore more vivid MI time coincide with actual ME time	Assumption that in an average population, there are no significant deviations in imagery execution time; however, this assumption omit the possibility of the typical subject have different imagery ability, which creates a research gap in this field
	dRT	[56]	The difference in reaction time (dRT) of ME task measured before MI training and after, or, post-train time difference between task trained with MI and random sequence task; also incorporate assumption about MI and ME connection	More adopted for sequence tasks than for instant tasks like grip
Implicit Motor Imagery				
Grip selection task	Skew driver task	[57,58]	Perspective judgment of gripping and real actual gripping have similar representation and, therefore, similar execution times	The same situation as with mental chronometry: grip selection task has not been used in a healthy population to investigate motor imagery ability variety
	Gasping and pouring from a container	[59]		
Motorically driven perceptual decisions	Hands Laterality Judgement Task (HLJT)	[60]	Subjects simulate the hand going from its current position to the stimulus’s orientation for comparison; therefore, the perceptual decision has a similar time to actual execution	-

**Table 3:** spTMS studies.

Paper	Stimulation Protocol	Task	Findings
[72]	The VAS was used to assess MI quality, whereas the MIQ-Revised was used to assess MI ability. During the MI task, a TMS pulse was applied to C3, and MEPs were measured in the abductor pollicis brevis (APB)	Piano playing of a simplified melody	The corticospinal excitability during MI + AO might be reflected by the VAS score, especially in complex MI movements
[73]	spTMS stimulus over M1 contralateral to the dominant hand while (1) congruent Action Observation (AO) + MI and (2) incongruent AO + MI; pure AO as a baseline	Rhythmical movements of index/little finger	AO and MI do not recruit the motor cortex to the same extent, rather, in both AO + MI settings, motor imagery alone can sufficiently explain the observed outcomes
[74]	spTMS stimulus was delivered to the left M1, the amplitudes of MEPs obtained during MI coupled with action observation (AOMI paradigm), independent action observation (AO), and independent MI were compared against a control condition	Basketball free-throw task (flexing and extending right wrist)	AOMI alone, but not independent MI or AO, enhanced corticospinal excitability
[75]	Corticomotor excitability (CE) during AOMI and AOMI + PAIN (muscle injection of hypertonic saline prior to AOMI task) sessions was assessed with TMS-evoked MEPs	Imagination and observation of index fingers’ abductions and adductions	The decrease in CE was counteracted by executing the AOMI task while being in pain, as evidenced by no change in TMS-MEPs during the AOMI + PAIN session
[76]	TMS delivery during MI over motor cortex of healthy human subjects, MEP measurements were taken for experimental and control groups	Wrist extension or flexion right after overt movement	Delivering TMS during MI is capable of inducing plastic changes in the motor system
[77]	Prior to the study, spTMS over C3 was used to define dominant TMS-evoked thumb movements; after training same spTMS setup was used to measure MEP during MI thumb movement	Thumb movement (flexion/extension) in the direction opposite to the predefined one	MEP amplitude during MI of thumb movements and measures of motor cortical adaptation following MI training have a strong positive connection
[78]	spTMS over C3 was used to determine dominant thumb movement + before and after of 5 training blocks of task completion	MI or ME of thumbs flexion/extension in the direction opposite to the dominant direction of the TMS-evoked thumb movements	MI can cause plastic changes similar to those observed while physical training, still it demands more training trials
[79]	TMS was used to define dominant, mean pre-train, and post-train directions of thumb movements	Isolated thumb extensions/flexions in a 90-degree angle (in the first experiment), 60 or 110-degree angle (in the second experiment) and from pre-defined TMS-evoked thumb direction	TMS-induced motions proportionately deviated in the trained direction -> MI causes use-dependent plasticity in the agonist muscle, which is accompanied by an increase in corticospinal excitability
[80]	Two groups—MI and MI + explicit instructions of avoiding overt movements; MEPs of spTMS over M1 during MI were compared for both groups	index finger-thumb opposition movements of a right hand	In the MI group, facilitatory effects were seen, while in the MI + explicit instructions group persisted inhibitory effects specific for the M1 contralateral to the hand performing the MI task

**Table 4:** rTMS studies.

Paper	Stimulation Protocol	Task	Findings
[83]	Suppressing neural activity of the dPMC, S1, and primary motor cortex (M1) with 1 Hz rTMS; spTMS over M1 MEPs to assess CE during kMI and vMI	abduction/adduction of right index finger	rTMS alters muscle-specific facilitation of CSE during kinesthetic but not visual motor imagery when applied to both dPMC and S1, but not M1; in particular, dPMC rTMS reduced CSE facilitation, whereas S1 rTMS increased it
[84]	Continuous inhibitory theta-burst TMS to the left IPL prior to MI-based implicit sequence learning (ISL) paradigm	Button presses with the non-dominant (left) hand	Mean dRT for the sham group was significantly greater than the mean dRT of the TMS group; IPL, and probably the visuospatial functions it mediates, are crucial for MI performance and consequently acquisition and learning of MI skill
[85]	Prior to the hands laterality judgment task (HLJT) and mental chronometry, subjects were given inhibitory rTMS stimulation to the left IPL	Hand laterality judgment	Inhibition of the left IPL impaired HLJT performance but not mental chronometry, demonstrating that the left IPL is involved in controllability and visual manipulation during MI
[86]	Prior to MI-based ISL paradigm training, subjects were given inhibitory rTMS over contralateral/ipsilateral PMC	Button presses with the non-dominant (left) hand	Similar mean dRT values across groups imply that MI-based learning is not affected by inhibition of the PMC -> effector-dependent encoding is not used in MI-based learning
[87]	Subjects with subacute stroke received LF rTMS + MI + Electrical stimulation ES/sham ES stimulations 5 days per week for 2 weeks; 1 Hz TMS was applied over contralesional hemispheric M1, ES over hemiplegic Upper Extremity (UE)	Arm movements include arm rising, elbow flexion and extension, wrist rotating, fist opening and releasing, and so on	Using LF rTMS + MI in combination with extra ES resulted in a better improvement in UE motor function of stroke subjects
[88]	rTMS + MI group was applied 1 Hz rTMS over the contralesional hemisphere combined with audio-led MI; the control group received the same rTMS parameters with audio-led relaxation; the LF-rTMS procedure was completed in ten 30-min sessions over the course of two weeks	Audio listening	LF-rTMS combined with MI significantly improved upper limb motor function and could be used to assist stroke patients in recovery of upper extremity motor function
[89]	One year post-stroke subjects were given 1 Hz rTMS (or sham rTMS for the control group) over non-stroke M1 coupled with BCI training for 3 weeks, followed by 3 weeks of BCI training alone	Gasping and lifting a cup via BCI	Motor improvements occurred in both groups, but only the TMS one demonstrated substantial inter-hemispheric inhibition changes in the intended direction, as well as increased relative ipsilesional cortex activation measured by fMRI; only the TMS group showed significant increases in BCI performance over time and adequate control of the virtual reality BCI paradigm.
[90]	Stroke patients of TMS-group received 12 sessions of 10 Hz rTMS stimulation over impaired M1 area while no stimulation was given to the control group; different BCI evaluation sessions were conducted afterward	Right and left hands tasks	In MI tasks, TMS improved BCI accuracy from 63.5 percent to 74.3 percent, and in ME tasks, it improved from 81.9 percent to 91.1 percent
